# Systematic Review on Plant‐Based Therapies for the Management of Sickle Cell Anaemia

**DOI:** 10.1155/anem/9936958

**Published:** 2026-08-10

**Authors:** Bot Yakubu Sunday, Swase Dominic Terkimbi, Okon Michael Ben, Sarad Pawar Naik Bukke, Idehen Iyore Charles, Herbert Izo Ninsiima, Adam Moyosore Afodun, Joseph Kibirige, Sara. I. A. Latif, Ejike Daniel Eze

**Affiliations:** ^1^ Department of Medical Laboratory Science, School of Allied Health Sciences, Kampala International University, Ishaka, Uganda, kiu.ac.ug; ^2^ Department of Pharmacy, Faculty of Health Sciences, Victoria University, Kampala, Uganda, vu.edu.au; ^3^ Department of Biochemistry, Faculty of Biomedical Sciences, Kampala International University, Ishaka, Uganda, kiu.ac.ug; ^4^ Department of Publication and Extension, Kampala International University, Ishaka, Uganda, kiu.ac.ug; ^5^ Department of Pharmaceutics and Pharmaceutical Technology, Kampala International University, Ishaka, Uganda, kiu.ac.ug; ^6^ Department of Public Health, Kampala International University, Ishaka, Uganda, kiu.ac.ug; ^7^ Department of Physiology, School of Medicine, Kabale University, Kabale, Uganda, kab.ac.ug; ^8^ Department of Anatomy and Cell Biology, Faculty of Health Sciences, Busitema University, Mbale, Uganda, busitema.ac.ug; ^9^ Faculty of Pharmacy, Shree S. K. Patel College of Pharmaceutical Education and Research, Ganpat University, Ganpat Vidyanagar, India, ganpatuniversity.ac.in; ^10^ Institute of Biomedical Research, Kampala International University, Ishaka, Uganda, kiu.ac.ug

**Keywords:** antioxidant mechanisms, antisickling, HbS polymerisation inhibition, phytochemicals, RBC membrane stabilisation

## Abstract

**Background:**

Sickle cell anaemia (SCA) is a hereditary haemoglobin disorder that continues to pose a significant public health burden worldwide. This systematic review aimed to synthesise evidence on plant‐derived therapies investigated for their antisickling, antioxidant and membrane‐stabilising effects in the management of SCA.

**Methods:**

A systematic search of PubMed, Scopus and Web of Science was performed according to PRISMA 2020 guidelines to identify studies (2010–2025) evaluating plant‐based interventions for SCA.

**Results:**

Thirty‐four studies published between 2010 and 2025 were included, with the majority (58.8%) published between 2018 and 2025, while 20.6% were published between 2010 and 2012. Most studies originated from Africa (58.8%), particularly Nigeria and the Democratic Republic of the Congo, followed by Asia (26.5%). *Carica papaya* was the most frequently investigated medicinal plant (21.2%), and leaves were the most commonly used plant part (39%). Strong antisickling activity was reported in 41.2% of studies, while 26.5% demonstrated inhibition of haemoglobin S (HbS) polymerisation and 17.6% reported membrane‐stabilising or antihaemolytic effects. Antioxidant activity accounted for 45.5% of all oxidative assessments. Overall, inhibition of HbS polymerisation was the most frequently reported mechanism of action, identified in 60.6% of the included studies.

**Conclusion:**

The evidence demonstrates that plant‐derived extracts and phytochemicals possess significant antisickling, antioxidant and membrane‐protective activity, supporting their potential as adjunct therapies for SCA. Nonetheless, standardisation of experimental protocols, toxicological evaluation and clinical trials are essential to advance towards clinical application.

## 1. Introduction

Sickle cell anaemia (SCA) remains one of the most significant inherited haemoglobinopathies affecting millions worldwide [1]. The disease burden is particularly high in sub‐Saharan Africa, where more than 75% of cases occur [2]. The disorder arises from a single‐point mutation in the β‐globin gene, resulting in haemoglobin S (HbS), which polymerises under hypoxic conditions. This polymerisation triggers erythrocyte sickling, loss of deformability, repeated cycles of haemolysis, vaso‐occlusion, chronic inflammation, oxidative stress and progressive organ dysfunction [3]. The complex pathophysiology of SCA involves interconnected processes including haemolysis, endothelial dysfunction, inflammation, oxidative injury and vascular occlusion [4].

In resource‐limited settings, SCA poses a significant clinical and socio‐economic burden due to inadequate access to diagnosis, treatment and long‐term care. Hydroxyurea, one of the most widely used disease‐modifying agents, has demonstrated significant benefits in increasing foetal haemoglobin levels and reducing vaso‐occlusive episodes. However, its application is limited by variable patient response, concerns about long‐term toxicity and irregular availability across low‐resource settings [5]. This has stimulated scientific interest in traditional medicinal plants, which are widely used across Africa, Asia and the Caribbean and other regions for managing SCA. Traditional medicine plays an important role in the management of sickle cell disease, with numerous plant‐based remedies used to alleviate pain and reduce haemolysis [6]. Over the last decade, experimental investigations have validated these traditional claims by demonstrating that numerous plant extracts possess antisickling, antipolymerisation, antioxidant, membrane‐stabilising, antihaemolytic and metabolic‐restorative properties [7]. These therapeutic actions are attributed to different phytochemical constituents including flavonoids, tannins, alkaloids, phenolic acids, anthocyanins, saponins, terpenoids and glycosides, which target multiple pathological pathways simultaneously [7].

Although evidence supporting the therapeutic potential of medicinal plants for sickle cell disease continues to grow, findings remain fragmented across studies with different experimental designs, extraction methods and mechanistic evaluations. Unlike previous reviews [6], which reported medicinal plants with antisickling activity or summarised ethnomedicinal applications. This review adopts a mechanistic and translational pharmacology perspective. Specifically, it synthesises experimental models, dose patterns, comparator use, phytochemical classes, plant parts, safety profiles and molecular mechanisms of action within a unified analytical framework. This approach provides conceptual and analytical advancement beyond earlier reviews by linking phytochemical composition and pharmacological mechanisms to translational potential.

## 2. Methods

### 2.1. Study Design

This systematic review followed the PRISMA 2020 framework throughout all phases of searching, screening and study selection [8]. The review was structured to identify experimental and preclinical evidence on plant‐based antisickling agents published between 2010 and 2025.

### 2.2. Information Sources

All included studies were published in peer‐reviewed journals indexed in international databases. A literature search was conducted across three major electronic databases: Web of Science, Scopus and PubMed.

### 2.3. Search Strategy

A combination of MeSH terms and free‐text queries was designed to capture all relevant studies. Core terms included ‘sickle cell anaemia’, ‘sickle cell disease’, ‘herbal medicine’, ‘plant extract’, ‘phytochemical’, ‘traditional medicine’ and ‘antisickling agent’. In databases such as Scopus and PubMed, the strategy further incorporated ethnobotanical keywords to capture research on traditional medicinal plants. Filters were applied to restrict results to English‐language articles and experimental designs involving human erythrocytes, animal models or mechanistic systems related to sickling. The initial search identified 296 records in Web of Science, 284 in Scopus and 30 in PubMed. After applying year and document‐type filters, the records were reduced to 100, 48 and 13, respectively, giving a combined total of 161 sources prior to duplicate removal.

### 2.4. Study Selection and Screening

**FIGURE 1:**
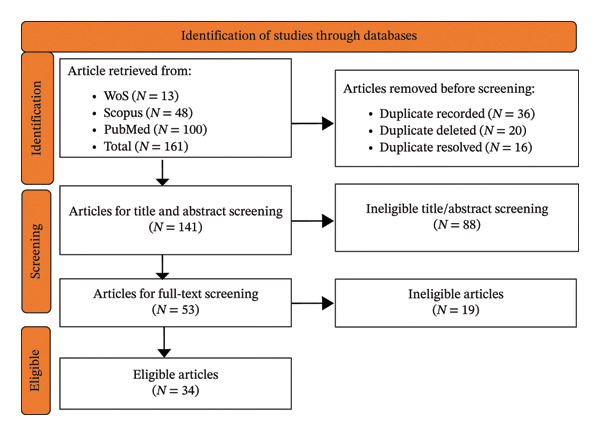
PRISMA flow chart.

All retrieved records were exported in CSV format and uploaded into Rayyan for systematic screening. Rayyan identified 38 duplicate records, of which 22 were removed, leaving 141 unique articles for screening. Two reviewers independently screened titles and abstracts in blinded mode. Of these, 46 studies were considered potentially eligible, and 81 were excluded based on predefined criteria. Fourteen screening conflicts occurred and were resolved through consensus, resulting in 53 articles proceeding to full‐text review. During full‐text assessment, 19 studies were excluded due to lack of experimental data, absence of antisickling evaluation, inaccessible full texts or methodological limitations. Four additional conflicts were resolved by consensus. Ultimately, 34 studies met the eligibility criteria and were included in the final synthesis (Figure 1).

### 2.5. Eligibility Criteria

Studies were eligible if they evaluated whole plant extracts, fractions, isolated phytochemicals or plant‐inspired synthetic analogues in experimental models of SCA. Synthetic analogues were included to assess the translational potential of plant‐derived molecular structures for antisickling drug development. Both in vitro and in vivo studies were considered eligible if they reported antisickling, antioxidant, membrane‐stabilising, haematological, polymerisation‐inhibiting or other relevant mechanistic outcomes. Studies published in languages other than English, review articles, commentaries, conference abstracts, studies lacking biological evaluation and articles unrelated to sickle cell disease were excluded.

### 2.6. Data Extraction

Data extraction was conducted independently by two reviewers using a structured Excel form. Extracted variables included study setting, year, country, plant species, plant part used, extraction procedure, type of extract, solvent system and experimental model. Biological outcomes such as antisickling activity, polymerisation inhibition, antioxidant capacity, membrane stabilisation, haematological changes and toxicity were recorded.

### 2.7. Quality Assessment

Quality assessment evaluated methodological rigour, including plant identification, extraction procedures, experimental models and validity of antisickling outcomes. Two reviewers independently assessed all studies, with disagreements resolved through consensus. Rayyan facilitated blinded screening, duplicate detection and conflict resolution to enhance consistency and minimise selection bias.

### 2.8. Data Synthesis

Data from the included studies were synthesised narratively due to differences in experimental models, plant species, extraction methods and outcome measures. Findings were organised into key themes, including phytochemical classes, mechanisms of antisickling activity and treatment outcomes across in vitro, ex vivo and in vivo models.

## 3. Results

### 3.1. Study Characteristics

**FIGURE 2:**
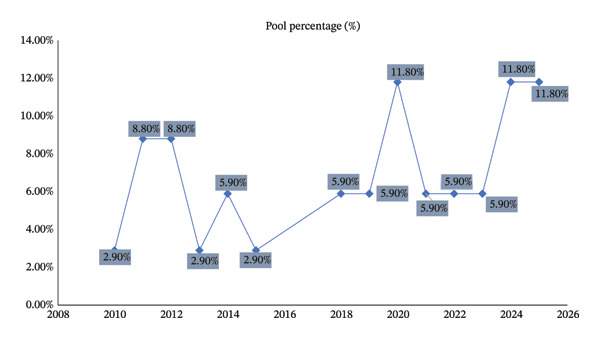
Temporal distribution of studies reported. The included studies were published between 2010 and 2025.

**FIGURE 3:**
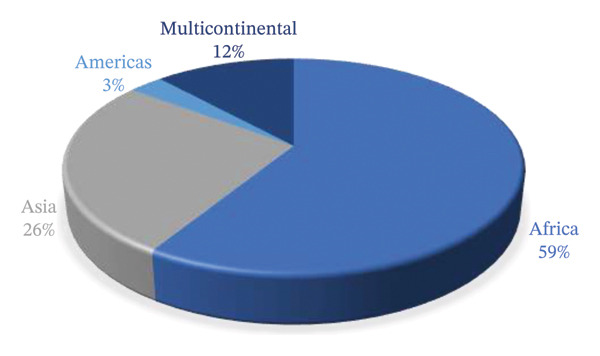
Regional contribution of studies included in the systematic analysis. Africa contributed 58.8% (*n* = 20) of the studies, followed by Asia (26.5%, *n* = 9), multicontinental collaborations (11.8%, *n* = 4) and the Americas (2.9%, *n* = 1).

The 34 included studies were published between 2010 and 2025, with most (58.8%) published between 2018 and 2025, while 20.6% were published between 2010 and 2012 (Figure 2). Most studies were conducted in Africa (58.8%), followed by Asia (26.5%). Multicontinental collaborations accounted for 11.8%, while the Americas contributed 2.9%. No study was conducted solely in Europe. Nigeria contributed the highest number of studies (44.1%), followed by the Democratic Republic of Congo (17.6%) and India (14.7%). Other countries, including Sri Lanka, Japan, Cameroon, Côte d’Ivoire, Senegal, Niger and the United States, had single or few contributions (Figure 3). Most studies used in vitro experimental designs with human HbSS erythrocytes. Common methods included the Emmel test, sodium metabisulphite‐induced sickling, HbS polymerisation assays, osmotic fragility and membrane stabilisation tests (Table 1).

**TABLE 1:** Study characteristics.

ID	Author (Year)	Country	Design	Model	Sample size	Method of extraction	Extract type	Solvent(s)	Safety/Toxicity
1	Anosike et al. (2019) [7]	Niger	In vitro experimental	Human SS blood	Not specified	In vitro	Aqueous and ethanolic	Water, ethanol	No deaths at 10,000 mg/kg
2	Chikezie (2011) [9]	Nigeria	In vitro experimental	HbSSM and HbSSF	Not specified	Maceration	Methanol + seed oil	Methanol	No toxicity
3	Asibor et al. (2024) [10]	Nigeria	In vitro experimental	HbSSM and HbSSF	Not specified	Not reported	Methanol + seed oil	Methanol	No toxicity
4	Chikezie et al. (2020) [11]	Japan	Experimental (in vivo)	hbS/S mice	*n* = 6	Boiled water extract	Broccoli sprout extract	Water	No adverse effects
5	Cyril‐Olutayo and Omotuyi (2022) [12]	DRC/Belgium	In vitro experimental	SS blood	7 batches	Decoction	Aqueous fractions	Water	High‐dose haemolysis
6	Dogara et al. (2022) [13]	Nigeria	In vitro experimental	SS erythrocytes	*n* = 3	Aqueous extraction	Crude aqueous	Water	No toxicity
7	Ejiofor et al. (2024) [14]	UK/Africa	Fractionation + metabolomics	HbSS RBCs	*n* = 3	Fractionation	Methanol fractions	DCM, MeOH, water	< 2% haemolysis
8	Elufioye et al. (2020) [15]	DR Congo	Survey + in vitro	HbSS RBCs	26 healers	Not reported	Aqueous extract	Water	No toxicity data
9	Anosike et al. (2018) [7]	India	Acute toxicity	Wistar rats	*n* = 6	14‐day observation	Herbal granules	None	No toxicity
10	Abdulmalik et al. (2011) [1]	USA/Saudi/Egypt	Medicinal chemistry	SS RBCs	Not specified	Synthetic compounds	Synthetic small molecules	DMSO	No toxicity data
11	José et al. (2022) [16]	USA/Nigeria	In vitro lab	HbSS RBCs	5 reps	Not stated	Aqueous extract	Water	No toxicity
12	Amponsah et al. (2024) [6]	India	In vitro experimental	SCD RBCs	Not specified	Not stated	Multiple extracts	Multiple	No toxicity
13	Kitadi et al. (2020) [17]	Nigeria	In vitro screening	HbSS RBCs	Not stated	Not stated	Crude extracts	MeOH, chloroform	Not evaluated
14	Kplé Tatiana et al. (2020) [18]	USA	Lab experimental	SS erythrocytes	Not specified	Short incubation	INN compounds	DMSO	No toxicity
15	Mishra et al. (2021) [19]	DR Congo	In vitro experimental	SS RBCs	Not stated	Not stated	Methanol extract	Acidified MeOH	Not assessed
16	Mishra et al. (2022) [20]	Nigeria	In vitro laboratory	HbSS blood	Not specified	7‐day extraction	Methanol and aqueous	MeOH, chloroform, water	Not assessed
17	Nurain et al. (2016) [21]	Nigeria	Ethnomedicinal + in vitro	SS blood	Not specified	Decoction + hydroethanol	Aqueous and hydro‐ethanol	Water, ethanol	Not assessed
18	Olori et al. (2023) [22]	India	In vitro experimental	HbSS RBCs	Not stated	Not stated	Crude extract	Petroleum ether, MeOH	Not assessed
19	Omar et al. (2025) [23]	Cameroon	In vitro experimental	SS RBCs	Not stated	Not stated	Aqueous and methanol	Water, MeOH	Not assessed
20	Afolabi et al. (2012) [5]	Côte d’Ivoire	In vitro experimental	SS RBCs	Not stated	2 h incubation	EZL and DZL	Hydroethanol, water	Not assessed
21	Omer et al. (2020) [24]	Nigeria	In vitro + in vivo	SS RBCs + rodents	Not stated	Aqueous‐methanol + fractionation	Crude + fractions	Multiple	LD50 > 8000 mg/kg
22	Olori et al. (2023) [22]	India	In vitro antisickling	HbS polymerisation	Not reported	Aqueous + chloroform	CAE and FSE	Water, chloroform	Not evaluated
23	Page et al. (2021) [8]	Nigeria	In vitro experimental	HbS polymerisation	*n* = 9	Aqueous extraction	Crude aqueous	Water	Not evaluated
24	Panda et al. (2019) [25]	Nigeria	Membrane assay	Osmotic fragility RBCs	Not stated	Fractionation	Leaf fractions	Multiple	Not evaluated
25	Pandey (2024) [26]	Senegal	In vitro experimental	SS and normal RBCs	Not stated	Successive extraction	CE, AqF, EAF	Hydroalcoholic, water, EA	Leucocyte proliferation
26	Patel et al. (2025) [27]	Nigeria	In vivo + ex vivo	Rats + SS RBCs	*n* = 6	Maceration	Crude ethanol	Ethanol	No toxicity
27	Abere et al. (2015) [2]	Nigeria	In vitro experimental	HbSS RBCs	Not stated	Soxhlet	Seed oil extract	*n*‐Hexane	Cardio‐friendly indices
28	Pauline et al. (2013) [28]	DR Congo	In vitro antisickling	HbSS RBCs	*n* = 3	Decoction + partition	L, MI, SF, LF, AF, OF	Water, MeOH, ether, CHCl3	No toxicity
29	Piccin et al. (2019) [29]	India	In vitro laboratory	SS RBCs	*n* = 3	Soxhlet	Multiple extracts	Multiple	No toxicity
30	Ranasinghe et al. (2012) [30]	Sri Lanka	Haemolysis assay	Healthy + dengue RBCs	Not stated	Cold‐water extraction	Aqueous extract	Water	No toxicity
31	Sahu et al. (2019) [31]	Nigeria	In vitro antisickling	Healthy + dengue RBCs	Not stated	Method of extraction	Aqueous extract	Solvent type	No toxicity
32	Salawu et al. (2025) [32]	India	In vitro experimental	Human + sickle RBCs	Not stated	Soxhlet	Ethanolic extract	Ethanol	No adverse effects
33	Sall et al. (2025) [33]	Nigeria	In vitro experimental	HbSS erythrocytes	1 donor	Cold maceration	Aqueous and methanol	Water, MeOH	Not reported
34	Mishra et al. (2021) [19]	DR Congo	In vitro experimental	SS erythrocytes	Triplicate assays	Cold percolation	Anthocyanin extract	Water, ethanol, ether	No toxicity

### 3.2. Distribution of Plant Species Evaluated for Antisickling Activity in the Included Studies

*Carica papaya* was the most common investigated plant, appearing in 21.2% of studies (Supporting File 1). Garcinia kola and polyherbal mixtures each at 9.1%. *Anacardium occidentale*, *Psidium guajava*, *Terminalia catappa*, *Solenostemon monostachyus*, *Ipomoea involucrata* and *Vernonia amygdalina* accounted for 6.1% each. The remaining plants (approximately 3.0% each) were reported only once. These include well‐known medicinal plants such as *Zingiber officinale*, *Hibiscus tiliaceus*, *Azadirachta indica*, *Moringa oleifera*, *Ceiba pentandra*, *Scoparia dulcis* and *Zanthoxylum* species, as well as many less commonly studied species such as *Hoslundia opposita*, *Buchhozia coriacea* and *Rauvolfia vomitoria*.

### 3.3. Distribution of Plant Parts Used Across Various Studies

**FIGURE 4:**
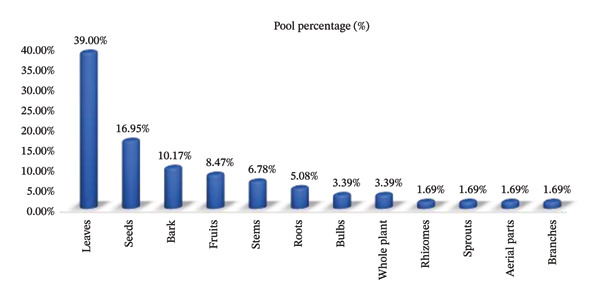
Distribution of plant parts used across various studies. Leaves accounted for 39.00% of the plant parts used, followed by seeds (16.95%), bark (10.17%), fruits (8.47%), stems (6.78%), roots (5.08%), bulbs (3.39%), whole plants (3.39%), rhizomes (1.69%), sprouts (1.69%), aerial parts (1.69%) and branches (1.69%).

Across the reviewed studies, various plant parts were used in antisickling preparations, with leaves being the most frequently reported, accounting for 39.0% of all plant materials (Figure 4). Seeds were the second most commonly used plant part (16.95%), followed by bark (10.17%), fruits (8.47%) and stems (6.78%). Roots accounted for 5.08%, while bulbs and whole plants each contributed 3.39%. Rhizomes, sprouts, aerial parts and branches were least represented, each accounting for 1.69% of the total.

### 3.4. Distribution of Phytochemical Classes Identified Across the Included Studies

**FIGURE 5:**
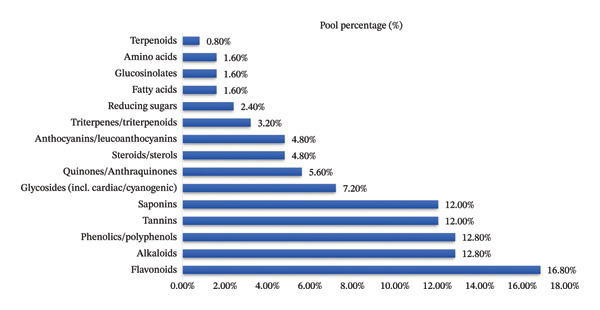
Distribution of phytochemical classes. Flavonoids (16.8%), alkaloids (12.8%), phenolic compounds (12.8%), tannins (12.0%), saponins (12.0%), glycosides (7.2%), quinones/anthraquinones (5.6%), steroids/sterols (4.8%), anthocyanins (4.8%), triterpenes (3.2%), reducing sugars (2.4%), fatty acids (1.6%), glucosinolates (1.6%), amino acids (1.6%) and terpenoids (0.8%) were identified across the included studies.

Flavonoids were the most commonly reported class (16.80%); this group included quercetin, kaempferol, rutin and anthocyanins [9, 12]. Alkaloids and phenolic compounds each accounted for 12.80%, while tannins and saponins each accounted for 12.00% [23]. Glycosides accounted for 7.20%, while quinones/anthraquinones accounted for 5.60% (Figure 5). Steroids/sterols and anthocyanins each accounted for 4.80%, and triterpenes (3.20%), reducing sugars (2.40%) and groups such as fatty acids, glucosinolates and amino acids (each 1.60%), while terpenoids accounted for 0.80%.

### 3.5. Experimental Model Utilisation and Antisickling Outcomes in Systematic Evidence

The most commonly used experimental approach was the direct sickling model (11/34; 32.4%), which evaluated untreated HbSS erythrocytes. Followed by chemically induced sickling models (9/34; 26.5%), where sickling was triggered using sodium metabisulphite (Na_2_S_2_O_5_), sodium dithionite (Na_2_S_2_O_4_) or nitrogen‐induced hypoxia. Polymerisation inhibition assays accounted for 3/34 studies (8.8%) and demonstrated suppression of HbS polymer formation and reduced turbidity. Osmotic fragility and membrane stability tests (2/34; 5.9%) reported increased resistance of erythrocytes to hypotonic lysis, while haemolysis assays (2/34; 5.9%) showed reduced red cell rupture. Survey‐linked studies (2/34; 5.9%) combined ethnomedicinal documentation with experimental validation. SAR, fractionation and metabolomics‐based approaches (2/34; 5.9%) identified phytochemicals associated with antisickling activity. In vivo, ex vivo and toxicity studies (4/34; 11.8%) reported tolerability of several extracts in animal models (Supporting File 2).

### 3.6. Distribution of Control and Comparator Use Reported Across Various Studies

Untreated HbSS/SS blood was the most frequently used control (29.4%) (Supporting File 3). Chemical positive controls, including vanillin, phenylalanine, p‐hydroxybenzoic acid, betulinic acid and 5‐HMF, were used in 26.5% of studies. Clinical comparators such as hydroxyurea, vitamin C and folic acid were reported in 17.6% of studies. Physiological controls, including PBS, normal saline and normal red blood cells (RBCs), were also used in 17.6%. Induction controls using sodium metabisulphite were reported in 11.8% of studies. Antioxidant or anti‐inflammatory comparators (quercetin, aspirin, arginine and indomethacin) were also used in 11.8%, while vehicle controls such as DMSO or distilled water were the least common (8.8%).

### 3.7. Distribution of Antisickling Effects Observed in the Reviewed Studies

Across the 34 studies, strong antisickling activity, characterised by reversal of sickling and restoration of normal RBC morphology, was reported in 41.2% of studies. Moderate antisickling effects were observed in 23.5%. Polymerisation inhibition was documented in 26.5% of studies, while membrane‐stabilising or antihaemolytic effects were reported in 17.6%. Weak or no antisickling activity was reported in 8.8% of studies, and 5.9% did not directly assess antisickling outcomes (Supporting File 4).

### 3.8. Distribution of Haematological Outcomes Across Antisickling Studies

Antioxidant activity was the most reported oxidative outcome, accounting for 45.5%. These extracts demonstrated free‐radical scavenging capacity through DPPH, FRAP, ABTS and hydroxyl radical assays. Reductions in oxidative injury markers were observed in 22.7% of studies, including decreased 8‐OHdG levels, reduced hepatic iron deposition, improved Fe^2+^/Fe^3+^ balance and lower methaemoglobin formation. Enzyme modulation contributed 18.2%, with increases in catalase, glucose‐6‐phosphate dehydrogenase (G6PD) and peroxidase supporting enhanced endogenous antioxidant defence. Mixed oxidative responses were reported in 13.6% of studies, where certain fractions exhibited both antioxidant and mild pro‐oxidant effects depending on dose. Metabolic pathway correction accounted for 9.1%, involving improvements in fatty acid and glucose–fructose metabolism and increased HbS solubility (Supporting File 5).

### 3.9. Proposed Mechanism Reported Across Various Studies

**FIGURE 6:**
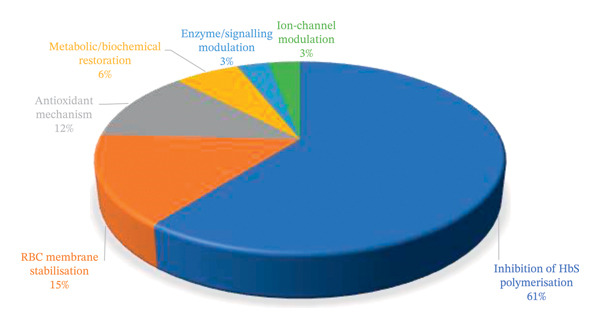
Distribution of dominant antisickling mechanisms across 34 studies. HbS polymerisation inhibition accounted for 60.6%, RBC membrane stabilisation for 15.2%, antioxidant activity for 12.1%, metabolic/biochemical restoration for 6.1% and enzyme/signalling pathway modulation and ion‐channel modulation for 3.0% each.

HbS polymerisation inhibition was the most reported mechanism, accounting for 60.6% (*n* = 20) of the included studies (Figure 6). RBC membrane stabilisation represented 15.2% (*n* = 5), followed by antioxidant mechanisms (12.1%, *n* = 4). Metabolic or biochemical restoration accounted for 6.1% (*n* = 2), while enzyme and signalling pathway modulation and ion‐channel modulation each contributed 3.0% (*n* = 1) of the studies.

## 4. Discussion

This review synthesises evidence on medicinal plants with antisickling activity, including experimental models, phytochemical classes and mechanisms of action. Most studies were conducted in Africa, particularly Nigeria and the Democratic Republic of Congo, which may be attributed to the high burden of sickle cell disease and the use of traditional medicinal plants. Several studies also originated from Asia, particularly India and Sri Lanka, suggesting the growing interest in medicinal plant‐based antisickling research in the region. This geographical distribution indicates the growing research activity in regions facing the burden of sickle cell, resulting in the application of traditional medicine.

In relation to the plant species investigated, *Carica papaya* was the most reported plant, although no single species dominated the antisickling activity across the studies included. This finding suggests that antisickling activity is not confined to a specific botanical lineage or chemical profile but may be present across different plant species and phytochemical families [12, 34]. The recurrent investigation of Garcinia kola, polyherbal formulations and several African medicinal plants may be due to their longstanding use in traditional medicine and their therapeutic value in SCD management [35, 36]. However, the limited replication of studies for most plant species indicates that many potentially valuable antisickling agents remain insufficiently explored. While the wide range of medicinal plants investigated expands therapeutic possibilities, it complicates cross‐study comparisons. Future research should prioritise standardised extraction methods, consistent experimental protocols and validation of promising plants across multiple studies.

Furthermore, the reported antisickling activity of these medicinal plants may be associated with the phytochemical constituents present in their extracts. Phytochemicals such as flavonoids, alkaloids, phenolics, tannins and saponins were reported across the investigated plants and may contribute to their antisickling effects. These phytochemicals are known to possess antioxidant, membrane‐protective and protein‐stabilising properties that target key pathophysiological mechanisms of SCD. Similar findings were reported in another systematic review, which identified significant antisickling activities associated with these phytochemical classes [37]. Their frequent co‐occurrence in plant extracts suggests that antisickling effects may result from synergistic interactions among multiple bioactive compounds [10]. For example, flavonoids and phenolics can reduce oxidative stress and stabilise erythrocyte membranes, while certain alkaloids may influence ion transport and cellular homeostasis [38]. Collectively, these findings highlight the potential contribution of plant secondary metabolites to the antisickling activity observed across different medicinal plants.

Despite these promising phytochemical characteristics, variations were observed in extraction methods, solvents and dosing regimens across the included studies. While most studies used aqueous or hydroethanolic extracts, others employed Soxhlet extraction, solvent partitioning and bioassay‐guided fractionation. This methodological heterogeneity limits direct comparisons of efficacy and may limit clinical translation. Nevertheless, antisickling activity was demonstrated across different experimental models. Direct sickling assays showed restoration of normal erythrocyte morphology, while chemically induced sickling models confirmed activity under hypoxic conditions [25]. Polymerisation assays provided mechanistic evidence by demonstrating inhibition of HbS polymer formation, a central event in sickling pathology [37]. Studies assessing osmotic fragility, haemolysis and membrane stability also reported improved erythrocyte integrity and resistance to cellular damage.

The findings from these experimental models provide insight into the mechanisms through which medicinal plants may exert their antisickling effects. Inhibition of HbS polymerisation was the most commonly reported mechanism (Supporting File 6). Several studies suggested that plant‐derived compounds may act through Schiff‐base formation, covalent interactions or stabilisation of the oxygenated (*R*) state of haemoglobin, thereby reducing the initiation of sickling [13]. Since HbS polymerisation is a key event in the pathogenesis of sickle cell disease, these findings may help explain the beneficial effects observed in the experimental studies.

In addition to HbS polymerisation inhibition, antioxidant activity emerged as another frequently reported mechanism. Oxidative stress is known to contribute to erythrocyte damage and disease progression in sickle cell disease, and its modulation may therefore have therapeutic relevance [39]. The antioxidant effects reported in the included studies may be mediated through free radical scavenging, modulation of catalase and G6PD activity and reduction of oxidative damage markers [5]. These mechanisms may contribute to reducing oxidative injury and enhancing erythrocyte stability, thereby complementing the antisickling effects associated with polymerisation inhibition [32]. Taken together, the evidence synthesised in this review suggests that medicinal plant extracts possess antisickling activity through different mechanisms, including inhibition of HbS polymerisation, membrane stabilisation and reduction of oxidative damage. However, most of the available evidence is derived from laboratory studies, with limited clinical data available. Further research should focus on preclinical evaluation, well‐designed clinical trials and standardisation of promising plant extracts to better establish their potential role in the management of sickle cell disease.

### 4.1. Study Limitations

1. Methodological heterogeneity existed across the included studies, with variations in experimental design, plant extraction methods, dosing units and antisickling assays, limiting comparability and precluding meta‐analysis.
2. Reporting quality was inconsistent, with many studies lacking essential details such as sample size justification, extraction yield, purity of isolated compounds or full methodological descriptions, reducing reproducibility.
3. The review protocol was not registered in PROSPERO because registration was not completed before the review commenced; however, the review followed a predefined protocol and PRISMA 2020 guidelines, and although the lack of registration may reduce methodological transparency and prospective verification, it does not compromise the validity of the review methods or findings.
4. Incomplete toxicological assessment was a major limitation; a considerable proportion of studies did not evaluate acute or chronic toxicity
5. Over‐reliance on in vitro data was evident, with few in vivo or ex vivo models and an absence of clinical trials, which constrains the translational relevance of the findings for patient care.
6. Geographical clustering of research predominantly in West and Central Africa may introduce regional bias and may not capture the full diversity of global ethnomedicinal plants

## 5. Conclusion

This systematic review shows that plant‐derived extracts and phytochemicals demonstrate consistent antisickling activity across multiple experimental models. Many agents were associated with inhibition of HbS polymerisation, restoration of erythrocyte morphology, antioxidant activity and improved membrane stability, supporting their potential as complementary therapeutic candidates for SCA. However, current evidence remains largely limited to in vitro and preclinical studies, with variability in extraction methods, dosing approaches and experimental models. Future research should prioritise phytochemical standardisation, pharmacokinetic and toxicological evaluation and well‐designed in vivo and clinical studies. Emerging computational approaches such as network pharmacology and molecular docking may also help identify key bioactive compounds, predict molecular targets and clarify multitarget mechanisms underlying antisickling activity. Integrating experimental and computational strategies will be essential for advancing plant‐derived antisickling agents towards clinically relevant therapies.

**Nomenclature**
μg/mL: Microgram per millilitre
ABTS: 2,2′‐azino‐bis(3‐ethylbenzothiazoline‐6‐sulfonic acid)
AF: Aqueous fraction
ART: Antiretroviral therapy
CEA: Crude ethanolic extract
CRISPR: Clustered regularly interspaced short palindromic repeats
DMSO: Dimethyl sulfoxide
DPPH: 2,2‐diphenyl‐1‐picrylhydrazyl
EA: Ethyl acetate
EAF: Ethyl acetate fraction
EZL: Ethanolic *Zanthoxylum leprieurii* extract
FRAP: Ferric reducing antioxidant power
G6PD: Glucose‐6‐phosphate dehydrogenase
HbAA: Normal adult haemoglobin
HbS: Haemoglobin S
HbSS: Homozygous sickle cell genotype
HBB: Helping babies breathe
HDL: High‐density lipoprotein
IC_50_: Half maximal inhibitory concentration
IDL: Intermediate‐density lipoprotein
Ig: Immunoglobulin
INN: Imidazole‐nitrone‐nucleophile compounds
LD_50_: Median lethal dose
LDL: Low‐density lipoprotein
LOX: Lipoxygenase
MeOH: Methanol
mg/kg: Milligram per kilogram
mg/mL: Milligram per millilitre
mM: Millimolar
MMA: Modified Michael acceptor compounds
N_2_: Nitrogen
Na_2_S_2_O_4_: Sodium dithionite
Na_2_S_2_O_5_: Sodium metabisulphite
Nqo1: NAD(P)H quinone dehydrogenase 1
PRISMA: Preferred reporting items for systematic reviews and meta‐analyses
RBC: Red blood cell
ROS: Reactive oxygen species
SAR: Structure–activity relationship
SCA: Sickle cell anaemia
SCD: Sickle cell disease
WHO: World Health Organisation
WoS: Web of Science

## Author Contributions

Bot Yakubu Sunday: conceptualisation, methodology, investigation, data curation and writing–original draft; Swase Dominic Terkimbi: methodology, formal analysis, validation and writing–review and editing; Okon Michael Ben: investigation, data curation and writing–review and editing; Sarad Pawar Naik Bukke: investigation, resources and writing–review and editing; Idehen Iyore Charles: formal analysis, visualisation and writing–review and editing; Herbert Izo Ninsiima: investigation and data curation; Adam Moyosore Afodun: resources and writing–review and editing; Joseph Kibirige: validation and visualisation; Sara. I. A. Latif: writing–review and editing; Ejike Daniel Eze: supervision, project administration and funding acquisition.

## Funding

This research received no funding from any agency in the public, commercial or not‐for‐profit sectors.

## Disclosure

The authors confirm that this manuscript is original and has not been published previously, in whole or in part, nor is it currently under consideration for publication by any other journal published by Wiley or any other publisher. The manuscript has been submitted exclusively to this journal in accordance with the journal’s submission requirements.

## Ethics Statement

This study is a systematic review based on previously published research and did not involve human participants, animals or the collection of new biological samples.

## Consent

The authors have nothing to report.

## Conflicts of Interest

The authors declare no conflicts of interest.

## Supporting Information

Additional supporting information can be found online in the Supporting Information section.

## Supporting information


**Supporting Information** Supporting File S1: Distribution of plant species evaluated for antisickling activity. Supporting File S2: Experimental model utilisation and antisickling outcomes in systematic evidence. Supporting File S3: Distribution of control and comparator use reported across various studies. Supporting File S4: Distribution of antisickling effects observed in the reviewed studies. Supporting File S5: Distribution of haematological outcomes across antisickling studies. Supporting File S6: Integrated summary of phytochemicals and key antisickling outcomes across included studies.

## Data Availability

All data generated or analysed during this study are included in this published article and its supporting files. Additional materials used during screening and extraction are available from the corresponding authors on reasonable request.
