# Supplementary material for: Systematic Review on Plant‐Based Therapies for the Management of Sickle Cell Anaemia
Source: Anemia. 2026 Aug 10;2026:9936958. doi: 10.1155/anem/9936958 (PMC13458021; doi:10.1155/anem/9936958)
Supplement: Supplementary file 1 [file ANEM-2026-9936958-s001.docx]

**Supplementary Files**

**Supplementary File 1: Distribution of Plant Species Evaluated for Antisickling Activity**

| **Plant (Scientific Name)** | **Common Name** | **Count** | **Percentage (%)** | **Reference** |
| --- | --- | --- | --- | --- |
| *Carica papaya* | Papaya / Pawpaw | 7 | 21.2% | (Sahu et al., 2019) |
| *Garcinia kola* | Bitter kola | 3 | 9.1% | (Salawu et al., 2025) |
| *Polyherbal mixtures* | Multiple species | 3 | 9.1% | (Kitadi et al., 2020) |
| *Anacardium occidentale* | Cashew | 2 | 6.1% | (Chikezie et al., 2020) |
| *Psidium guajava* | Guava | 2 | 6.1% | (Chikezie, 2011 |
| *Terminalia catappa* | Tropical almond | 2 | 6.1% | (Chikezie, 2011) |
| *Solenostemon monostachyus* | SolMon | 2 | 6.1% | (Afolabi et al., 2012) |
| *Ipomoea involucrata* | Ipocrata | 2 | 6.1% | (Afolabi et al., 2012) |
| *Vernonia amygdalina* | Bitter leaf | 2 | 6.1% | (Afolabi et al., 2012) |
| *Flueggea virosa* | – | 1 | 3.0% | (Souleymane et al., 2023) |
| *Kigelia africana* | Sausage tree | 1 | 3.0% | (Souleymane et al., 2023) |
| *Brassica oleracea (sprouts)* | Broccoli (Sulforaphane) | 1 | 3.0% | (Panda et al., 2019) |
| *Hibiscus tiliaceus* | Sea hibiscus | 1 | 3.0% | (Borive Amani et al., 2025) |
| *Zingiber officinale* | Ginger | 1 | 3.0% | (Salawu et al., 2025) |
| *Allium sativum* | Garlic | 1 | 3.0% | (Salawu et al., 2025) |
| *Alchornea cordifolia* | Christmas bush | 1 | 3.0% | (Adeniyi et al., 2022) |
| *Woodfordia fruticosa* | Gujaratis | 1 | 3.0% | (Mishra et al., 2022) |
| *Hoslundia opposita* | African mint bush | 1 | 3.0% | (Olori et al., 2023) |
| *Catharanthus roseus* | Madagascar periwinkle | 1 | 3.0% | (Olori et al., 2023) |
| *Rauvolfia vomitoria* | African serpentwood | 1 | 3.0% | (Olori et al., 2023) |
| *Lantana camara* | Wild sage | 1 | 3.0% | (Olori et al., 2023) |
| *Euphorbia hirta* | Asthma-plant | 1 | 3.0% | (Olori et al., 2023) |
| *Ocimum basilicum* | Basil | 1 | 3.0% | (Tshilanda et al., 2014) |
| *Xylopia aethiopica* | African pepper | 1 | 3.0% | (Cyril-Olutayo & Omotuyi, 2022) |
| *Nauclea latifolia* | African peach | 1 | 3.0% | (Cyril-Olutayo & Omotuyi, 2022) |
| *Olax subscorpioidea* | Olax | 1 | 3.0% | (Cyril-Olutayo & Omotuyi, 2022) |
| *Mangifera indica* | Mango | 1 | 3.0% | (Cyril-Olutayo & Omotuyi, 2022) |
| *Buchhozia coriacea* | Musk tree | 1 | 3.0% | (Cyril-Olutayo & Omotuyi, 2022) |
| *Securidaca longipendunculata* | Violet tree | 1 | 3.0% | (Kitadi et al., 2020) |
| *Khaya senegalensis* | African mahogany | 1 | 3.0% | (Kitadi et al., 2020) |
| *Zanthoxylum zanthoxyloides* | Chewing stick | 1 | 3.0% | (Kitadi et al., 2020) |
| *Cajanus cajan* | Pigeon pea | 1 | 3.0% | (Kitadi et al., 2020) |
| *Scoparia dulcis* | Sweet broomweed | 1 | 3.0% | (Abere et al., 2015) |
| *Azadirachta indica* | Neem | 1 | 3.0% | (Verma et al., 2018) |
| *Helianthus annuus* | Sunflower | 1 | 3.0% | (Verma et al., 2018) |
| *Terminalia avicennioides* | Black plum / Terminalia tree | 1 | 3.0% | (Sahu et al., 2019) |
| *Moringa oleifera* | Drumstick tree | 1 | 3.0% | (Omer et al., 2020) |
| *Mucuna flagellipes* | Cowitch | 1 | 3.0% | (Ejiofor et al., 2024) |
| *Ceiba pentandra* | Silk cotton tree | 1 | 3.0% | (José et al., 2022) |
| *Rubia cordifolia* | Indian madder | 1 | 3.0% | (Mishra et al., 2021) |
| *Bacopa monnieri* | Brahmi | 1 | 3.0% | (Pandey, 2024) |
| *Telferia occidentalis* | Fluted pumpkin | 1 | 3.0% | (Atabo et al., 2014) |
| *Justicia secunda* | Blood root | 1 | 3.0% | (Kitadi et al., 2020) |
| *Zanthoxylum heitzii* | Fagara heitzii | 1 | 3.0% | (Pauline et al., 2013) |
| *Zanthoxylum leprieurii* | African satinwood | 1 | 3.0% | (Kplé Tatiana et al., 2020) |
| Synthetic MMA compounds | Not a plant | 1 | 3.0% | (Afolabi et al., 2012) |

The results show that Carica papaya was the most frequently investigated plant (21.2%), followed by Garcinia kola and polyherbal mixtures (each 9.1%). A second group of plants including *Anacardium occidentale*, *Psidium guajava*, *Terminalia catappa*, *Solenostemon monostachyus*, *Ipomoea involucrata*, and *Vernonia amygdalina* each appeared in 6.1% of studies.

**Supplementary File 2**.**: Experimental Model Utilization and Antisickling Outcomes in Systematic Evidence**

| **Model Category** | **Description** | **Examples** | **N** | **%** | **Reference** |
| --- | --- | --- | --- | --- | --- |
| Direct sickling models | SS blood used without chemical induction | Emmel test; untreated HbSS erythrocytes; HbSSM/HbSSF RBCs | 11 | 32.4% | (Afolabi et al., 2012; Souleymane et al., 2023) |
| Chemically induced sickling models | Na₂S₂O₅, Na₂S₂O₄, or N₂-induced hypoxia | Na₂S₂O₅-induced sickling; Na₂S₂O₄-induced hypoxia; 100% nitrogen anoxia | 9 | 26.5% | (Panda et al., 2019; Salawu et al., 2025) |
| Polymerization inhibition assays | HbS polymer formation inhibition | HbS polymerization assay using Na₂S₂O₅; turbidity inhibition tests | 3 | 8.8% | (Adeniyi et al., 2022; Afolabi et al., 2012) |
| Osmotic fragility / membrane stability | RBC membrane resistance to osmotic stress | Osmotic fragility (0.3–0.9 g/100 mL NaCl) | 2 | 5.9% | (Chikezie, 2011; Chikezie et al., 2020) |
| Hemolysis assays | Hemolysis inhibition in human erythrocytes | Hemolysis inhibition using healthy and dengue RBCs | 2 | 5.9% | (Elufioye et al., 2020; Kitadi et al., 2020) |
| Survey-linked in-vitro assays | Ethnobotanical/ethnomedicinal survey + in-vitro testing | Survey + Na₂S₂O₄-induced SS RBC sickling | 2 | 5.9% | (Kitadi et al., 2020) |
| SAR / fractionation / metabolomics | Chemical profiling linked with antisickling assays | Bioassay-guided fractionation + SS RBC testing; SAR + antisickling assays | 2 | 5.9% | (Mishra et al., 2021; Ranasinghe et al., 2012) |
| In-vivo / ex-vivo / toxicity models | Animal toxicity studies + ex-vivo SS RBC evaluation | Wistar rats (acute toxicity); mice (hbS/S); rat 30-day toxicity; ex-vivo SS RBC assays | 4 | 11.8% | (José et al., 2022; Pandey, 2024) |

Direct sickling models accounted for 32.4% of studies, followed by chemically induced sickling models (26.5%). Polymerisation inhibition assays represented 8.8%, while osmotic fragility and hemolysis assays each accounted for 5.9%. Survey-linked studies and SAR/fractionation approaches each represented 5.9%, and in-vivo/toxicity models accounted for 11.8%.

**Supplementary File 3: Distribution of Control and Comparator Use reported Across various studies**

| Control / Comparator Category | Description | Examples | N | % | Reference |
| --- | --- | --- | --- | --- | --- |
| Untreated HbSS / SS blood controls | Baseline sickle cell condition used to compare reversal of sickling and morphological restoration. | Untreated SS blood; SS blood alone; HbSS untreated | 10 | 29.4% | (Omer et al., 2020; Ranasinghe et al., 2012) |
| Chemical positive controls | Known antisickling agents used to benchmark plant/extract potency. | p-HBA; Vanillin; Betulinic acid; 5-HMF; Phenylalanine | 9 | 26.5% | (Imaga et al., 2013; Verma et al., 2018) |
| Standard clinical comparators | Clinically relevant agents used in SCD management to validate biological relevance of results. | Hydroxyurea; Vitamin C; Folic acid | 6 | 17.6% | (Nurain et al., 2016; Salawu et al., 2025) |
| Normal/physiological controls | Non-sickle blood or physiological solutions used to assess normal cell behavior or baseline hemolysis. | PBS; Normal saline; Normal RBCs | 6 | 17.6% | (Adejumo et al., 2011; Ejiofor et al., 2024) |
| Induction controls | Chemical agents used to artificially induce sickling as a stress or challenge model. | Na₂S₂O₅-induced sickled RBCs; 2% Na₂S₂O₅ | 4 | 11.8% | (Chikezie, 2011; Chikezie et al., 2020) |
| Antioxidant / anti-inflammatory comparators | Compounds tested to determine whether antioxidant or anti-inflammatory pathways contribute to antisickling effects. | Quercetin; Arginine; Aspirin; Indomethacin | 4 | 11.8% | (Abdulmalik et al., 2011; Tshilanda et al., 2014) |
| Vehicle / procedural controls | Solvents or carriers used to ensure test effects are not due to the vehicle. | DMSO; MF diet feed; Distilled water | 3 | 8.8% | (Adeniyi et al., 2022; Salawu et al., 2025) |

Untreated HbSS/SS blood was the most common baseline control (29.4%), followed by chemical positive controls such as vanillin, phenylalanine, p-HBA, and betulinic acid (26.5%). Clinical comparators, including hydroxyurea, vitamin C, and folic acid, were used in 17.6% of studies, similar to physiological controls such as PBS and normal saline (17.6%). Induction controls using Na₂S₂O₅-sickled RBCs were reported in 11.8% of studies. Antioxidant or anti-inflammatory comparators (quercetin, aspirin, indomethacin) also accounted for 11.8%, while vehicle controls were least common (8.8%).

**Supplementary File 4: Distribution of Antisickling Effects Observed in the Reviewed Studies**

| **Outcome Category** | **Description** | **Examples From Studies** | **N** | **%** | **Reference** |
| --- | --- | --- | --- | --- | --- |
| Strong antisickling & RBC normalization | Marked reversal of sickling, restoration of biconcave shape, high normalization rates (>70%). | 75–95% normalization; 87.2–93% inhibition; 78% of plants ≥70%; 81.37% reversal; >70% NRC (MI, AF). | 14 | 41.2% | (Anosike et al., 2019) |
| Moderate antisickling activity | Partial inhibition or reversal of sickling (50–70%). | 50–67% normalization (MMA compounds); 66% reversal; 58–61% extract activity; 64–68% inhibition. | 8 | 23.5% | (Pandey, 2024) |
| Polymerization inhibition | Inhibition or delay of HbS polymer formation, turbidity reduction, polymer growth suppression. | INN-312 (15–95%); INN-298 (18–80%); strong inhibition in multiple plant extracts; SolMon & Ipocrata polymerization inhibition; 84.79% inhibition at 30s (P. guajava). | 9 | 26.5% | (Atabo et al., 2014) |
| Membrane stabilization / anti-hemolytic effect | Reduced hemolysis, improved membrane integrity, decreased K⁺ efflux. | T. catappa & A. occidentale fractions stabilizing; membrane stabilization with reduced K⁺ efflux; EAF improved stability vs control. | 6 | 17.6% | (Afolabi et al., 2012) |
| Low or no antisickling effect | Minimal or absent antisickling activity. | Broccoli-sulforaphane diet: no improvement; some petroleum ether/n-butanol fractions inactive; P. guajava destabilizing except n-hexane. | 3 | 8.8% | (Cyril-Olutayo & Omotuyi, 2022) |
| Not directly assessed | Study focused on toxicity or reported known effects without testing antisickling activity. | Toxicity-only study; papaya studies reporting prior data but not tested here. | 2 | 5.9% | (Omer et al., 2020) |

Reversal of sickling and restoration of normal RBC morphology were the most frequently reported outcomes (41.2%), followed by polymerisation inhibition (26.5%) and moderate antisickling effects (23.5%). Membrane-stabilising or anti-hemolytic effects were reported in 17.6% of studies. Low or no activity was observed in 8.8%, while 5.9% of studies did not directly evaluate antisickling effects.

**Supplementary File 5: Distribution of Hematological Outcomes Across Antisickling Studies**

| **Oxidative Stress Outcome Category** | **Description** | **Examples From Studies** | **N** | **%** | **Reference** |
| --- | --- | --- | --- | --- | --- |
| Antioxidant activity demonstrated | Extracts showed free-radical scavenging or reducing power. | DPPH IC50 = 6.6–32.25 μg/mL, FRAP increase, ABTS IC50 = 0.068 mg/mL, 91.24% DPPH at 75 µg/mL | 10 | 45.5% | (Anosike et al., 2018; Pandey, 2024) |
| Reduction in oxidative damage markers | Lowering of molecular or biochemical markers of oxidative injury. | Decrease 8-OHdG; ↓ hepatic iron, decrease, MHb, improved Fe²⁺/Fe³⁺ ratio | 5 | 22.7% | (Vona et al., 2021) |
| Enzyme modulation | Extracts altered endogenous antioxidant enzymes. | Increase G6PD, decrease Catalase, increase, Peroxidase | 4 | 18.2% | (Afolabi et al., 2012) |
| Mixed / partial oxidative responses | Antioxidant and pro-oxidant effects depending on dose/fraction. | F7 strong antioxidant, F1/F8 weak or pro-oxidant, mixed catalase effects | 3 | 13.6% | (Tshilanda et al., 2014) |
| Metabolic pathway correction | Extracts corrected metabolic pathways linked to oxidative imbalance. | Fatty acid/glucose–fructose pathway correction, increase HbS solubility | 2 | 9.1% | (Borive Amani et al., 2025; Salawu et al., 2025) |

Antioxidant activity was the most frequently reported oxidative outcome (45.5%). Reductions in oxidative damage markers were reported in 22.7% of studies, while enzyme modulation accounted for 18.2%. Mixed oxidative responses were observed in 13.6%, and metabolic pathway correction in 9.1% of studies

**Supplementary File 6: Integrated Summary of Phytochemicals and Key Antisickling Outcomes Across Included Studies**

| **Category** | **Plants (Examples)** | **Phytochemicals** | **Key Results** | **Reference** |
| --- | --- | --- | --- | --- |
| Strong antisickling activity (polyphenol- and flavonoid-rich plants) | *Flueggea virosa*, *Kigelia africana*, *Hibiscus tiliaceus*, *Garcinia kola*, *Ocimum basilicum*, *Scoparia dulcis*, *Carica papaya* | Flavonoids, tannins, saponins, phenolics, anthocyanins, quinones | Potent inhibition and reversal of sickling; normalization of RBC morphology; strong polymerization inhibition; dose-dependent effects; supports traditional use. | (Souleymane et al., 2023),(Borive Amani et al., 2025) |
| Antioxidant + Anti-inflammatory + Cytoprotective extracts | *Hibiscus tiliaceus* (F7), *Woodfordia fruticosa*, *Terminalia avicennioides*, *Bacopa monnieri*, *Zanthoxyllum heitzii*, *Rubia cordifolia* | Rutin, kaempferol derivatives, polyphenols, phenolic acids, flavonoids, anthocyanins | Strong DPPH/FRAP/ABTS activity; ROS reduction; anti-inflammatory effects; membrane stabilization; synergistic antioxidant–antisickling activity. | (Asibor et al., 2024; Salawu et al., 2025) |
| High-potency specific bioactive compounds | *Alchornea cordifolia* (Quercitrin), *Ocimum basilicum* (Butyl stearate), *Zanthoxyllum* species (various alkaloids) | Quercitrin, butyl stearate, alkaloids | Identified compounds show superior antisickling and membrane-stabilizing activity compared to standard controls (e.g., quercetin). | (Adeniyi et al., 2022; Tshilanda et al., 2014) |
| Broad ethnomedicinal validation / multi-plant screens | 23-plant panel including *Alchornea cordifolia*, *Alternanthera bettzickiana*, *Annona senegalensis*, *Justicia secunda*, *Hypoxis angustifolia* | Mixed secondary metabolites | High antisickling activity across many African medicinal plants; majority >70% normalization; strong validation of traditional use. | (Kitadi et al., 2020; Sahu et al., 2019) |
| Synthetic antisickling agents (very high potency) | MMA series (MMA-202, MMA-204), INN series (INN-312, INN-298) | Synthetic azolylacryloyl derivatives | Up to 90-fold more potent than vanillin; sustained P50 shift; improved metabolic stability; increased deoxy-HbS solubility; rational drug-design success. | (Pauline et al., 2013) |
| Fraction-dependent or solvent-dependent activity | *Garcinia kola*, *Ceiba pentandra*, *Rubia cordifolia*, *Carica papaya* (multiple extracts), *Terminalia catappa* | Phenols, flavonoids, cardiac glycosides, saponins | Distinct fractions show different strengths (e.g., methanol = strongest antisickling; acetone = strongest antioxidant; aqueous = highest XO inhibition). | (Chikezie, 2011; Verma et al., 2018) |
| Membrane stabilizers / osmotic fragility improvement | *Anacardium occidentale*, *Psidium guajava*, *Terminalia catappa* | Fatty acid methyl esters, pentacosane, dibutyl phthalate | Petroleum ether, ethyl acetate, and n-hexane fractions show strong membrane stabilization; dose- and time-dependent protection. | (Chikezie et al., 2020) |
| Plants with metabolic pathway correction | Broccoli (Sulforaphane), *Mucuna flagellipes* | Sulforaphane, oleic/linoleic acids, vitamins A/E/K, zinc | Corrected fatty acid and glucose–fructose pathways; improved Fe²⁺/Fe³⁺ ratio; reduced heme overload and liver necrosis. | (Imaga et al., 2013; Panda et al., 2019) |
| Strong multi-mechanistic activity (antisickling + antioxidant + membrane effects) | *Zanthoxyllum heitzii*, *Terminalia avicennioides*, *Moringa oleifera* | Polyphenols, saponins, alkaloids, anthocyanins | Fruit or polar extracts strongest; high antioxidant power; membrane-protection; multiple mechanisms contribute to antisickling. | (Kplé Tatiana et al., 2020) |
| Validated safe & non-toxic formulations | Sharpunkhadi polyherbal mix; multiple plant extracts with LD₅₀ > 5000 mg/kg | Mixed plant metabolites | High safety margins; no mortality; mild or no toxicity; supports potential clinical translation. | (Patel et al., 2025; Salawu et al., 2025) |

This table summarizes 34 studies by linking plant phytochemicals to their biological effects. Flavonoids, tannins, saponins, phenolics, and anthocyanins showed the strongest antisickling activity, including inhibition of HbS polymerization, reversal of sickling, and improved red cell stability. Several extracts also demonstrated antioxidant and anti-inflammatory effects. Isolated compounds such as quercitrin and butyl stearate showed high activity, while synthetic analogues (MMA and INN series) demonstrated enhanced antisickling effects.
